# The dignified approach to care: a pilot study using the patient dignity question as an intervention to enhance dignity and person-centred care for people with palliative care needs in the acute hospital setting

**DOI:** 10.1186/s12904-015-0013-3

**Published:** 2015-04-09

**Authors:** Bridget Johnston, Jan Pringle, Marion Gaffney, Melanie Narayanasamy, Margaret McGuire, Deans Buchanan

**Affiliations:** Sue Ryder Care Centre for the Study of Supportive, Palliative and End of Life Care, School of Health Sciences, The University of Nottingham, Queen’s Medical Centre, Nottingham, NG7 2HA, England UK; School of Education, Social Work and Community Education, University of Dundee, C.2.16 Carnelley Building, Dundee, DD1 4HN, Scotland UK; Royal Victoria Hospital, Roxburghe House, Jedburghe Road, Dundee, DD2 1SP, Scotland UK; NHS Tayside Headquarters, Ninewells Hospital and Medical School, Dundee, DD1 9SY, Scotland UK; Ninewells Hospital, NHS Tayside, Dundee, DD1 9SY, Scotland UK

**Keywords:** Palliative care, Dignity, Acute care, Mixed methods

## Abstract

**Background:**

Providing person-centred, dignity-conserving care for hospitalised patients is central to many healthcare policies and essential to the provision of effective palliative care. The Patient Dignity Question (PDQ) “*What do I need to know about you as a person to take the best care of you that I can*?” was designed from empirical research on patients’ perceptions of their dignity at end of life to help healthcare professionals (HCPs) understand the patient as a person.

**Methods:**

This mixed method pilot study was designed to inform a larger multisite study in the future. It tests the hypothesis that the PDQ intervention could be used to enhance a more person-centred climate for people with palliative care needs in the acute hospital setting, and provide evidence regarding its acceptability. Outcome measures pre and post intervention Person-centred Climate Questionnaire – patient version (PCQ-P), and the Consultation and Relational Empathy (CARE) measure; PDQ feedback questionnaires were used for all participants post intervention, in addition to qualitative interviews.

**Results:**

30 patients, 17 HCPs, and 4 family members participated. Results showed a positive correlation between higher PCQ-P scores and higher CARE scores, indicating that the PDQ can make improvements to a person-centred environment and levels of empathy perceived by patients. Individual results from the PCQ-P and the CARE indicated overall improvements in the majority of fields. The PDQ supported disclosure of information previously unknown to HCPs, has implications for improving person-centred care. Positive results from PDQ feedback questionnaires were received from all participants.

Qualitative findings indicated patients’ appreciation of staff (*Attributes and attitudes*), that patients wanted staff to have awareness of them (*Know me as a person*), take the time to talk, and work flexibly, to allow for patient individuality (*Time and place*).

**Conclusion:**

The PDQ has potential to improve patients’ perceptions of care, and HCP attitudes. Furthermore, it was well received by participants. The PDQ could be incorporated into clinical practice for the care of palliative care patients in the acute setting to the benefit of personalized and dignified care.

Further research using the PDQ across wider geographical areas, and more diverse settings, would be beneficial.

**Electronic supplementary material:**

The online version of this article (doi:10.1186/s12904-015-0013-3) contains supplementary material, which is available to authorized users.

## Background

Providing person-centred, dignity conserving care for hospitalised patients is central to many health care policies. It is also an approach to care that is essential to providing effective palliative care. The Patient Dignity Question (PDQ) “*What do I need to know about you as a person to take the best care of you that I can*?” is a question that has been designed from empirical research on patients’ perceptions of their dignity at the end of life to help HCPs understand the patient as a person.

This pilot study was conceived to help inform the design of a future large scale multicentre trial. It follows an earlier feasibility study [1], which helped clarify whether study elements were viable. These included recruitment procedures, willingness of participants to be randomised and characteristics of the proposed outcome measure. This use of feasibility studies is supported [2,3]. The findings of the feasibility study suggested that it was possible to carry out a full-scale pilot study. It led the researchers to incorporate another necessary patient outcome measure- the Consultation and Relational Empathy (CARE) measure, which highlights aspects relating to levels of empathy. This, along with the Person-centred Climate Questionnaire (PCQ-P), allows a thorough assessment of dignified care, since empathy is pertinent to dignity, and a nationally recognised core standard of care [4]. Thus, including both of these measures allows dignified care to be thoroughly explored.

In line with recommended use of pilot studies by Whitehead *et al.* and Arain *et al.* [2,3], this current study represents a smaller version of a planned larger scale study by focussing on the processes that will be used in the future study. This includes the allocation of participants to the PDQ intervention and the follow-up assessments using outcome measures.

Since findings from pilot studies can contribute to the final analysis carried out during the main study [2], this paper provides analysis and discussion of the main results stemming from the pilot study.

Specifically, this pilot study aimed to test the use of the Patient Dignity Question as a brief intervention to foster a more person-centred climate by promoting a therapeutic relationship between HCPs and their patients. The question was developed during extensive work by Chochinov and colleagues at the University of Manitoba, Canada, to help deliver dignity conserving care to people at the end of life [5]. The PDQ reflects the association between a sense of dignity, and patients feeling known as individuals, rather than in relation to their diagnoses.

It was hypothesised that the PDQ could be used as an intervention to enhance care for people with palliative needs in the acute care setting. This study was conceptualised to test that hypothesis, and provide evidence regarding the use and acceptability of the PDQ; it builds on an earlier feasibility study [6].

### Aim

The primary aim of this study, as a smaller version of a future large scale trial, was to explore the effectiveness of the PDQ as an intervention to improve person-centred care. A secondary aim was to determine the overall acceptability of the PDQ for patients, families and staff.

**Primary Hypothesis:** the PDQ as an intervention will result in more positive post-intervention scores for person-centred care and empathy compared to baseline scores.

**Secondary Hypothesis:** the PDQ as an intervention will be acceptable to patients, families and staff.

## Methods

### Study design

A mixed method before and after design was used. This method is particularly effective for testing and evaluating complex interventions which are patient-centred and individualised [7], since it allows data from a variety of sources to be combined. Such an approach can strengthen a study by allowing triangulation of findings, to ensure that conclusions are drawn from several diverse sources of evidence, thereby increasing legitimacy [7]. This is particularly valuable for pilot studies, where new areas are being investigated. As clarified in the background section, a pilot study was conducted in order to assess the test of the methods, procedures and procedures to be used on a larger scale in the full scale trial. It has also been argued that is important to report pilot study results, particularly before proceeding to a full-scale trial [8] as pilot study findings can contribute to the final analysis [2]. Data was therefore collected using standard outcome measures as well as qualitative methods. The pilot study was pragmatic in that it intended to evaluate the effectiveness of the PDQ in real life clinical practice, i.e. a busy acute hospital setting.

The PCQ-P outcome measure [9] was used to determine patients’ views of the environment in which they were being cared for. This 17 item, self-report instrument evaluates the extent to which the climate of the healthcare setting is perceived to be person-centred (see Additional file 1). It has been validated for use in the hospital setting.

The CARE [10] is a consultation process measure developed by Stewart Mercer and colleagues in the Departments of General Practice at Glasgow and Edinburgh Universities. It is based on a broad definition of empathy in the context of a therapeutic relationship within the care environment, and has been evaluated and accredited for use in measuring the human aspects of clinical encounters.

The PDQ patient and family feedback questionnaire and the PDQ healthcare provider feedback questionnaire [5] were also used as outcome measures to explore participant views about the dignity question. These questionnaires are currently in the process of being validated, but were considered appropriate tools to use, since they relate specifically to the use of the PDQ, and can give valuable feedback about acceptability. Permission to use the questionnaires was granted by Professor Chochinov.

Qualitative data was provided by semi-structured interviews and open responses from the feedback questionnaires. Standard demographic measures were recorded (gender, age, diagnosis, time since diagnosis etc.) and, to assess stage of disease further, the Palliative Performance Scale [11] and Palliative Prognostic Index [12] were also recorded for each patient.

### Setting

Participants were recruited from acute care wards in one teaching hospital in the East of Scotland. The hospital has a specialist palliative care team (led by consultants in palliative medicine, and with clinical nurse specialists) to offer support and advice across the hospital. Patients are assessed by ward staff (doctors, nurses and allied health professionals) who will provide general palliative care if this is what the patients require. If patients warrant more specialist palliative care, patients are referred to the hospital specialist palliative care team. Patients may be transferred to the acute palliative care unit in the hospital or one of two local hospices. Id there needs require this. Care is thus provided according to patient needs, and goals of care are agreed with the patient. Therefore, palliative care may be the only mode of treatment or an extra layer of support for those receiving active treatment for concurrent acute issues. Pallliative care needs mean those with incurable illnesses who have unmet needs, these may be along the physical, social, psychological and/or spiritual continuum.

### Participants

Inclusion criteria: adult patients with any level of palliative care needs (as determined by their hospital consultant and care team), their nominated family member(s), and healthcare professionals responsible for their care.

Exclusion criteria: those not meeting the inclusion criteria; those too ill, frail or cognitively impaired to take part.

### Steering group

A Project Management (Steering) Group was convened to oversee the running and management of the project and to comment on the data generated throughout the project. This group was composed of HCPs, the research team, and a patient representative. The group met quarterly for the 12 months duration of the project.

### Ethics

This study received approval from The East of Scotland Research Ethics Committee, (13/ES/0033) and NHS Tayside R&D (2013ON09).

No personal or identifiable information was used beyond the immediate research sites, where all data was held securely. During the transcription of interviews, participants were assigned a numerical value, and these have been used for any quotes in results sections of reports or publications.

### Recruitment

Information about the study and a training session were provided for all HCPs involved in the study prior to the recruitment of patients; this included information on how to use the PDQ in clinical practice. Patients were approached by HCPs who were involved in their care and, if interested in participating, were also invited to identify a family member to take part in the study. All participants were asked to provide written consent.

### Study procedures

Patients were asked the PDQ “*what do I need to know about you as a person to take the best care of you that I can*?” by a HCP, who took a written note of what was being said. A summary of their response was passed to the researcher who then checked that the patient was satisfied with the summary. If the patient indicated that they wished for amendments to be made to the summary, these were made by the researcher. Once accuracy was confirmed by the patient, the summary was typed up and the researcher requested the patients’ permission to display it in medical and nursing records. The patient was asked to complete the PCQ-P and CARE questionnaires before the PDQ was asked. The PDQ feedback questionnaire was given to the patient to fill out any time on the same day as the PDQ had been asked. The PCQ-P and CARE questionnaires were given to the patient to fill out again 48 hours after the PDQ summary had been displayed in nursing and medical records. Patients were also invited to take part in an audio recorded interview.

A HCP who was caring for each patient participant, and who was available and willing to take part, was identified by the researcher, and asked to complete the HCP PDQ feedback questionnaire. Family members identified by the patient were also asked to complete the PDQ feedback questionnaire and were invited to take part in an interview at least 48 hours after the PDQ summary had been displayed.

### Quantitative analysis

Quantitative data were provided by patient, family, and HCP feedback questionnaires. Pre and post PDQ patient feedback was elicited from two questionnaire designs: The Person-centred Climate questionnaire –patient version (PCQ-P) and The Consultation and Relational (CARE) Measure questionnaire. General results were tabulated and collapsed where appropriate. Scores from each item on the two questionnaires were summated to give an overall PCQ-P score out of 102 and an overall CARE score out of 50, both pre and post PDQ. These summated scores were used in Wilcoxon Signed Rank Tests to explore post-PDQ- intervention effect.

A further questionnaire collected patient and family feedback on opinions about the PDQ intervention, data of which were also tabulated. From the PDQ feedback questionnaires, patients or family members’ responses to 6 feedback questions with yes/no answers were converted to percentages. HCPs were asked to rate the effect of the PDQ on eight items upon a 7-point scale. Summation of the eight outcomes formed a composite PDQ Responsiveness Score (PRS), giving a global quantitative measure of the PDQ’s effect, and allowing inferential statistical exploration. This involved independent-sample t-tests and analysis of variance tests. All descriptive and inferential statistical tests were carried out using IBM SPSS Statistics 21 computer software [13].

### Qualitative analysis

Qualitative data provided by the semi-structured interviews and open responses from the feedback questionnaires were analysed using Framework Analysis [14]. The framework approach was developed in the UK by social policy researchers, specifically for applied or policy relevant qualitative research. The objectives of the investigation are typically set in advance and shaped by the information requirements of the funding body. The framework analytical process is explicit, and designed to be viewed and assessed by people other than the primary analyst, thereby leaving a clear ‘audit trail’; the use of charts tracks how data progresses from transcripts to themes in a transparent manner, following a sequential rather than concurrent approach. It allows a case and a theme based approach to be combined, looking within and across cases within the same analysis [15]. Framework Analysis (FA) therefore provides an applied, pragmatic approach to analysis, rather than aligning to a specific research paradigm. This involved familiarisation (data immersion and review of all data), development of a theoretical framework (thematic analysis and charting of themes and sub-themes into matrices, indexing (fitting of framework to data with modification of themes to be made if fit is not accurate), summarising (transforming detailed material into brief, accurate summaries, which can be related back to transcripts), and synthesising of data (mapping and checking back to original context, making further amendments if necessary).

Themed categories were identified by the research team based on the research objectives and questions. The matrix tables were devised using Microsoft Word to aid the organisation of the data. For reliability and validity (trustworthiness) purposes, two research team members cross checked codes derived from the interviews.

## Results

### Recruitment

We recruited 30 patients, who nominated 4 family members to participate; 17 HCPs also participated (total 51 participants).

### Demographic information

Patient participants ranged from 38–86 years, with a mean age of 65.4 yrs. Family members were either spouses or siblings, aged 61–71 yrs (mean 69 yrs). HCP participants had been qualified for between 2 months and 29 years, and ranged in age from 22–50 yrs.

See Tables 1, 2 and 3 below for further details.

**Table 1:** **Patient demographics (N = 30)**

**Variable**	**N**	**%**	**M**	**SD**
Male	14			
Female	16			
Age (Years)			65.4	10.8
Palliative Performance			54.3	11.4
Palliative Prognostic Index			2.6	2.1
Diagnosis:				
Missing	2	6.7		
Advanced abdominal cancer	1	3.3		
Anal cancer	1	3.3		
Anorectal cancer	1	3.3		
Bowel cancer	1	3.3		
Chronic Kidney Disease	2	6.7		
Lung cancer	5	16.7		
Metastatic breast cancer	1	3.3		
Metatistic bladder cancer	1	3.3		
Myeloma	2	6.7		
Oesophageal cancer	1	3.3		
Ovarian cancer	6	20		
Pancreatic cancer	2	6.7		
Peritoneal cancer	1	3.3		
Prostate cancer	1	3.3		
Renal cancer and lung cancer	1	3.3		
Vulval melanoma	1	3.3

**Table 2:** **Family member demographics (n = 4)**

**Participants**	**Age range**	**Gender**	**Relationship to patient**
Family members	61-	1 F	Husband: 2
	71 yrs	2 M	Sister: 1
	Mean 69 yrs	1 NK	Not known: 1

**Table 3:** **Health care provider demographics (N = 17)**

**Variable**	**N**	**%**	**M**	**SD**
**Male**	3	17.6		
**Female**	13	76.5		
**Missing demographics**	1	5.9		
**Age (years)**			34.1	10.3
**Profession:**				
Charge nurse	3	17.6		
Doctor	4	23.5		
FY2	1	5.9		
FY1	2	11.8		
Oncologist	1	5.9		
Other	1	5.9		
Staff nurse	7	41.2		
Pharmacist	1	5.9		
Physiotherapist	1	5.9		
**No. of years qualified**			8.0	9.5
**No. of years in this post**			2.1	1.8
**Has had previous palliative care experience**	6	35.3		
**PDQ Responsiveness Score (PRS)**			39.4	9.6

### Quantitative results

#### Results from the PCQ-P

Baseline and post-intervention scores were compared using the Person-centred Climate Questionnaire.

Overall increases were the case for 82% of the PCQ-P questionnaire items (14 out of 17 items) compared to decreases. Greatest increases were seen in patients’ perceptions of staff being easy to talk to (+43.3%; −13.3%); that there was something nice to look at (+40%; −16.7); that people talked about ordinary things rather than just illness (+36.7%; −16.7); and where they felt they could get ‘that little bit extra’ (+33.3%; −23.3%). Other overall increases included thinking of the climate as a place where the patient relies on receiving the best care (+26.7%; −10%), where the patient feels in safe hands (+30%; −16.7%), and where the patient feels welcome (+26.7%; −16.7%). Reductions were seen in patients’ perceptions of staff taking notice of what they say (−26%; +20%) and where the staff make a little extra effort on their behalf (−23.3%; +13.3%). No change scores ranged from 43.3%-63.3%. See Table 4 for further details.

**Table 4:** **Effect of PDQ on patients’ PCQ-P scores (N = 30)**

**PCQ-P question: I experience the climate here as a place …**	**Decrease**	**No change**	**Increase**	**Missing**
	**N (%)**	**N (%)**	**N (%)**	**N (%)**
Where the staff are knowledgeable	6 (20.0%)	18 (60.0%)	6 (20.0%)	0 (0%)
Where I rely on receiving best care	3 (10.0%)	19 (63.3%)	8 (26.7%)	0 (0%)
Where I feel in safe hands	5 (16.7%)	16 (53.3%)	9 (30%)	0 (0%)
Where I feel welcome	5 (16.7%)	17 (56.7%)	8 (26.7%)	0 (0%)
Where It is easy to talk to staff	4 (13.3%)	13 (43.3%)	13 (43.3%)	0 (0%)
Where the staff take notice of what I say	8 (26.7%)	16 (53.3%)	6 (20.0%)	0 (0%)
Where the staff come quickly when I need help	5 (16.7%)	19 (63.3%)	6 (20.0%)	0 (0%)
Where the staff use a language I can understand	6 (20.0%)	17 (56.7%)	7 (23.3%)	
Which is neat and clean	4 (13.3%)	19 (63.3%)	7 (23.3%)	
Where the staff have time for the patients	4 (13.3%)	18 (60.0%)	7 (23.3%)	1 (3.3%)
Where there is something nice to look at	5 (16.7%)	14 (46.7%)	11 (36.7%)	0 (0%)
Which feels homely even though I am in an institution	5 (16.7%)	16 (53.3%)	8 (26.7%)	1 (3.3%)
Where it is possible to get unpleasant thoughts out of my head	6 (20%)	15 (50.0%)	9 (30.0%)	0 (0%)
Where people talk about ordinary things not just illness	5 (16.7%)	14 (46.7%)	11(36.7%)	0 (0%)
Where the staff make a little extra effort on my behalf	7 (23.3%)	19 (63.3%)	4 (13.3%)	0 (0%)
Where I have choices, for example what to wear	6 (20.0%)	17 (56.7%)	7 (23.3%)	0 (0%)
Where I can get “that little bit extra”	7 (23.3%)	13 (43.3%)	10 (33.3%)	0 (0%)

**Figure 1:**
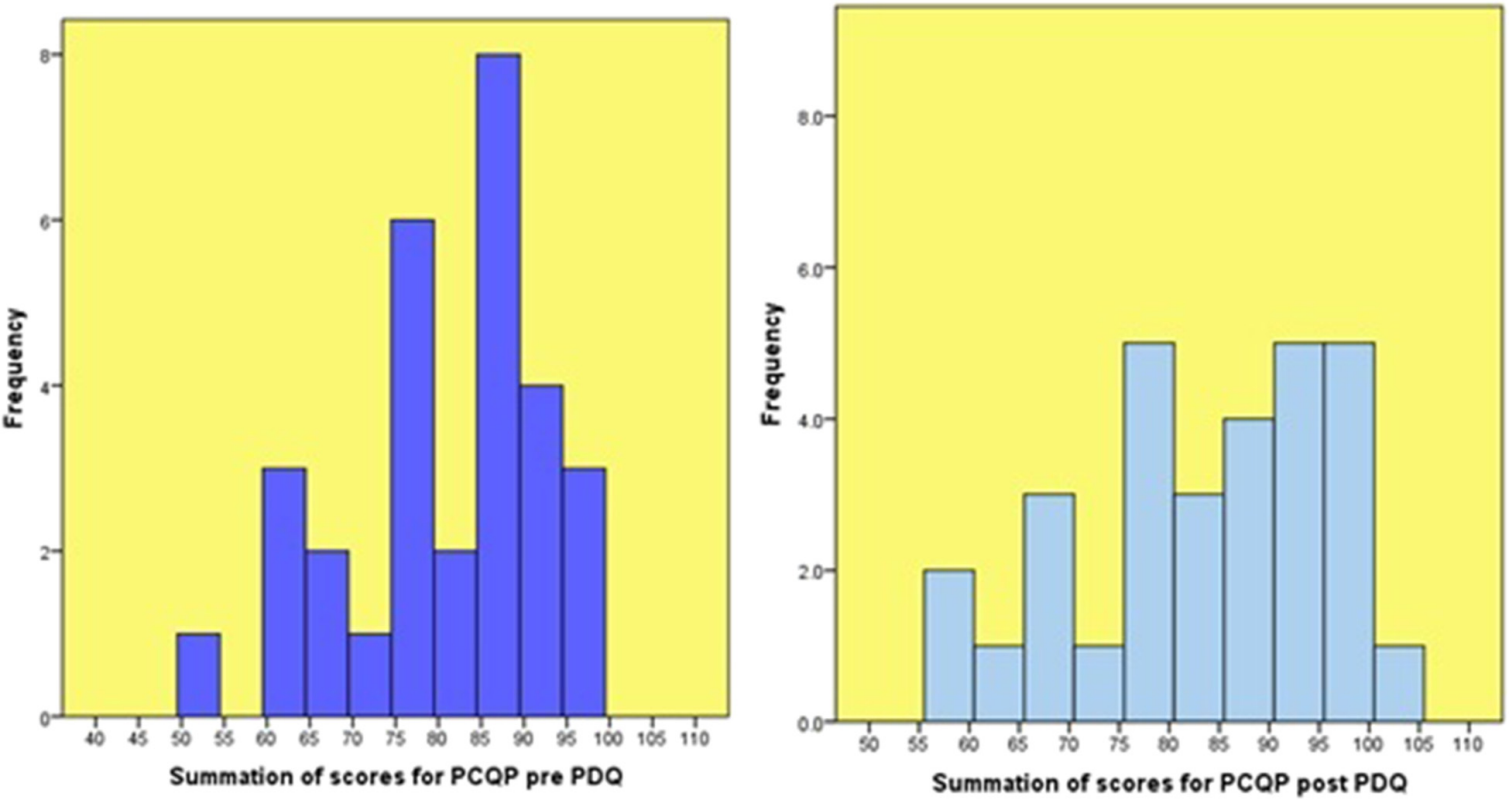
**Graphs 1 and 2- Histograms of summation of scores for PCQ-P pre and post PDQ.**

The median was chosen as the best measure of central tendency since the data did not have normal distribution (Figure 1). This suggests that the mean value may not be ideal to focus on as the central value of distribution [11]. Table 4 shows that the median value increased post PDQ. This was also the case for the lower and upper quartiles. Furthermore, the highest post PDQ PCQ-P score was 101, out of a maximum of 102. In addition, the minimum post PDQ PCQ-P score has increased from pre PDQ. This increase in the lowest score for PCQ-P suggests that overall perceptions of dignified care (as relating to person-centred care) improved after the PDQ intervention.

#### CARE scores

For several items on the CARE questionnaire, there were significantly more patients whose scores increased compared to those whose scores decreased. These included helping the patient to take control (+36.7%; −13.3%), the professional being interested in the patient as a whole person (+36.7%; −13.3%), the professional being positive (+33.3%; −10%) and professional making a plan of action with the patient (+33.3%; − 13.3%). In contrast, there were slightly more patients whose scores decreased than increased for the issue of the healthcare team explaining things clearly (−20%; +16.7%). No change scores ranged from 43.3%-66.7%.

Further details relating to CARE scores are given in Table 5 below.

**Table 5:** **Effect of PDQ on patients’ CARE scores (N = 30)**

**CARE measure question: How was your healthcare team at…**	**Decrease**	**No change**	**Increase**	**Missing**
	**N (%)**	**N (%)**	**N (%)**	**N (%)**
Making you feel at ease?	4 (13.3%)	20 (66.7%)	6 (20.0%)	0 (0%)
Letting you tell your “story”?	5 (16.7%)	18 (60.0%)	4 (13.3%)	3 (10%)
Really listening?	2 (6.7%)	20 (66.7%)	8 (26.7%)	0 (0%)
Being interested in you as a whole person?	5 (16.7%)	14 (46.7%)	11 (36.7%)	0 (0%)
Fully understanding your concerns?	5 (16.7%)	18 (56.7%)	6 (20.0%)	1 (3.3%)
Showing care and compassion?	5 (16.7%)	17 (56.7%)	8 (26.7%)	0 (0%)
Being positive?	3 (10.0%)	17 (56.7%)	10 (33.3%)	0 (0%)
Explaining things clearly?	6 (20.0%)	19 (63.3%)	5 (16.7%)	0 (0%)
Helping you to take control?	4 (13.3%)	13 (43.3%)	11 (36.7%)	2 (6.7%)
Making a plan of action with you?	4 (13.3%)	16 (53.3%)	10 (33.3%)	0 (0%)

Palliative Performance Scale (PPS) scores for patients ranged from 30-70% (mean 54.3%; SD 11.4); Palliative Prognostic Index (PPI) scores ranged from 0–7.0 (mean 2.6; SD 2.1).

Histograms were produced to display the distribution of summated PCQ-P scores pre and post PDQ intervention (Figure 1). The graphs in this Figure show that the data is skewed and not normally distributed. Comparing the histogram in Graph 1 with the histogram in Graph 2, some lower scores have higher frequencies post PDQ. However, overall there is an accumulation of higher scores in Graph 2.

Summated CARE scores pre and post PDQ (Table 6) reveal that the median score remained the same post-PDQ. However, CARE scores did increase post-PDQ for the lower and upper quartiles of data. The maximum summated score did not increase post-PDQ; however, 50 is the highest score possible, suggesting that an excellent level of empathetic care was already present before the PDQ intervention. The minimum summated CARE score increased by 5 post-PDQ.

**Table 6:** **Summation of scores for PCQ-P and CARE pre and post PDQ**

**Variable**	**N**	**Minimum**	**Maximum**	**Range**	**Lower quarter**	**Median**	**Upper quartile**
Summation of scores for PCQ-P pre PDQ	30	52	96	44	74.00	83.00	89.25
Summation of scores for PCQ-P post PDQ	30	58	101	43	74.75	85.00	94.25
Summation of scores for CARE pre PDQ	30	20	50	30	29.75	43.00	47.25
Summation of scores for CARE post PDQ	30	25	50	25	35.50	43.00	48.00

**Figure 2:**
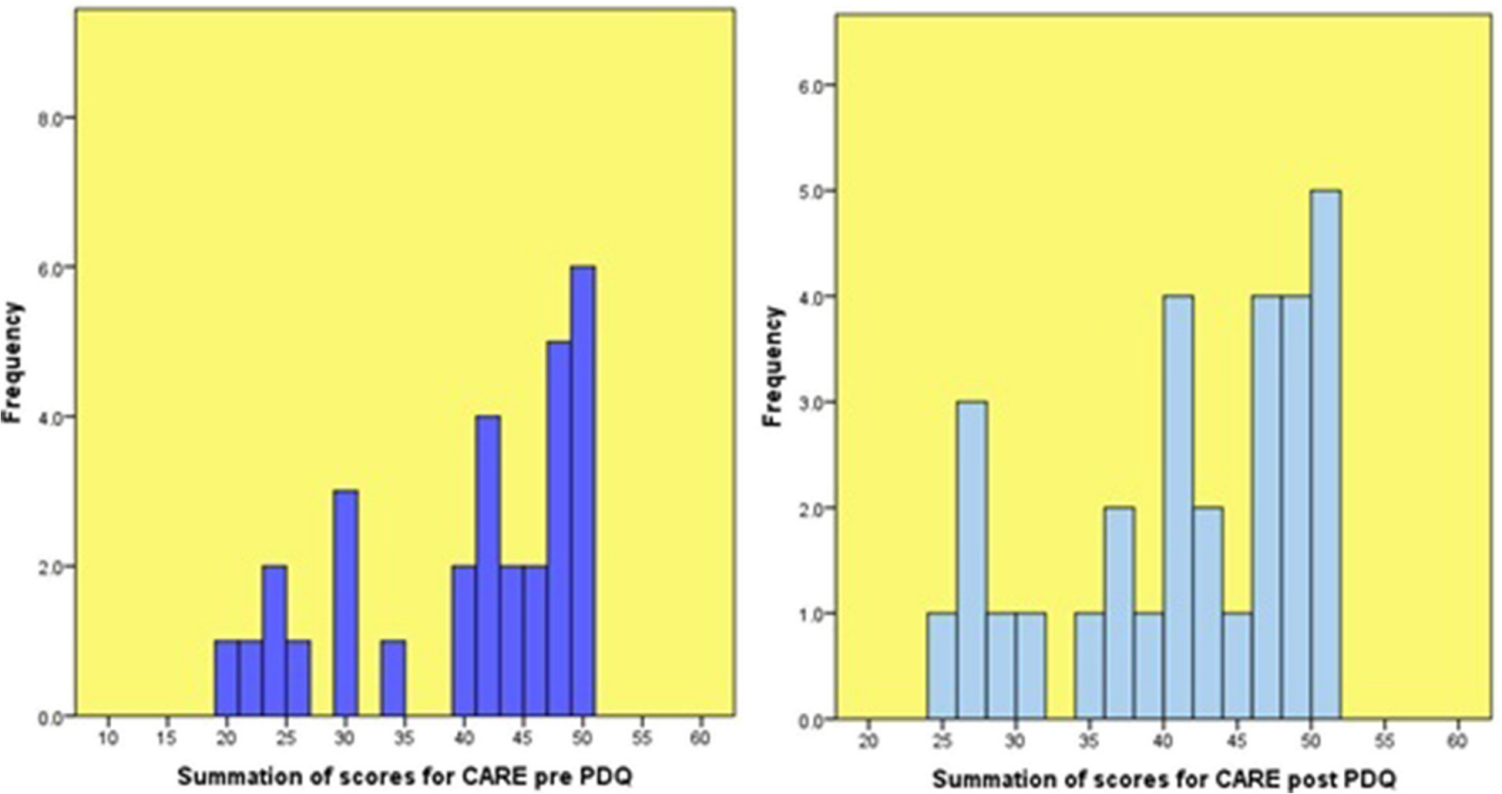
**Graphs 3 and 4- Histograms of summation of scores for CARE pre and post PDQ.**

Two further histograms were produced to display the distribution of summated CARE scores pre and post PDQ intervention (Figure 2). As with the PCQ-P data, the data is skewed, which again, indicates that the distribution is not normal. Although there were higher frequencies of some lower summated scores post-PDQ generally, Graph 4 displays higher frequencies for the larger scores compared to Graph 3.

Beyond descriptive analyses, inferential statistics were explored to make comparisons between the two groups. Non-parametric Wilcoxon Signed Rank Tests were carried out because, as Graphs 1, 2, 3 and 4 show (Figures 1 and 2), the data is skewed and not normally distributed. With large samples (those greater than 30), parametric techniques are often robust enough to tolerate the violation of normal distribution [16]. However, given that our sample for patients was 30, it was deemed more appropriate to pursue non-parametric techniques.

The Wilcoxon Signed Rank Test did not reveal a statistically significant reduction in summated PCQ-P scores following the PDQ intervention, *z* = −1.59, *p* = 0.11 (>0.05). However, as shown in Table 6 the median score for PCQ-P increased from pre-PDQ (*Md =* 83.00) to post-PDQ (*Md* = 85.00).

Similarly, a Wilcoxon Signed Rank Test was carried out to see whether CARE score differences between the two time periods (pre and post PDQ) were statistically significant. The test failed to show statistical significance from pre- PDQ (*Md* = 43.00) to post-PDQ (*Md* = 43.00), *z* = −0.85, *p* = 0.4 (>.05).

The relationship between summated PCQ-P score post PDQ and summated CARE score post PDQ was investigated using Spearman’s rho correlation coefficient. There was strong, positive correlation between the two variables, *r* = .67, *n* = 30, *p* = 0.000 (<.0005). This shows that higher PCQ-P scores post-PDQ are associated with higher CARE scores post PDQ.

#### Results from the patient feedback survey

From the patient feedback survey, all patients (100%) indicated that the information was accurate and all supported this information being put on their charts. A high percentage of patients (83.3%) felt that the information was important for HCPs to know. Although less felt that the PDQ would affect the way that the HCP gave care (63.3%), this figure represents well over half of patients. This positive patient feedback is further strengthened by the fact that a large majority were willing to recommend the PDQ intervention to others (93.3%). See Table 7.

**Table 7:** **PDQ patient feedback survey (N = 30)**

**Feedback survey: The PDQ**	**No. Responded yes**	**%**
Was accurate	30	100
Can be put on chart	30	100
Would like copy	10	33.3
Information is important for HCP	25	83.3
Would affect the way HCP give care	19	63.3
Recommend to others	28	93.3

#### Healthcare provider response

Healthcare providers’ responses to the PDQ were generally very positive. See Table 8 for details. As is evident in Table 8, from the ‘slightly agree’ and ‘agree or strongly agree’ columns, the majority of health care participants concurred about the positive impact of the PDQ on their attitude (87.5%), care (62.5%), respect (62.6%), empathy (81.3%), connectedness (81.3%), and satisfaction (62.6%) with the care they were able to offer. Further analyses of HCPs’ responses were carried out and since the PRS scores were normally distributed, parametric techniques were conducted. Independent-samples t-tests were conducted to compare the PDQ Responsive Score (PRS) and HCP characteristics.

**Table 8:** **Effect of PDQ on health care providers (based on N = 17 responses)**

**Healthcare provider response to PDQ**	**Strongly disagree or disagree**	**Slightly disagree**	**Neutral**	**Slightly agree**	**Strongly agree or agree**
	**N (%)**	**N (%)**	**N (%)**	**N (%)**	**N (%)**
Learn something new from PDQ	3 (18.8%)	2 (12.5%)	1 (6.3%)	4 (25.0%)	7 (43.8%)
Was emotionally affected by PDQ	2 (12.5%)	0 (0%)	5 (31.3%)	5 (31.3%)	5 (31.3%)
PDQ influenced attitude	1 (6.3%)	0 (0%)	2 (12.5%)	4 (25.0%)	10 (62.5%)
PDQ influenced care	1 (6.3%)	0 (0%)	6 (37.5%)	2 (12.5%)	8 (50.0%)
PDQ influenced respect	2 (12.5%)	0 (0%)	5 (31.3%)	3 (18.8%)	7 (43.8%)
PDQ influenced empathy	2 (12.5%)	0 (0%)	2 (12.5%)	4 (25.0%)	9 (56.3%)
PDQ affected connectedness	1 (6.3%)	0 (0%)	3 (18.8%)	4 (25.0%)	9 (56.3%)
PDQ affected satisfaction caring for patient	1 (6.3%)	0 (0%)	6 (37.5%)	1 (6.3%)	9 (56.3%)

Results show that there were no significant difference in scores based on gender or previous palliative care experience. One-way between groups analysis of variance (ANOVA) tests were also conducted and revealed no significant differences between PRS scores and characteristics including age groups, number of years the HCP had been qualified, and number of years in current healthcare role.

### Qualitative results

In addition to open responses from feedback questionnaires, 10 patients and 2 family members took part in qualitative interviews at least 48 hours after the intervention. Following familiarisation with the interview transcripts and feedback comments (Framework Analysis, stage 1), second stage frameworks were drawn up for each group of participants separately (i.e. patients, family members, and HCPs). This allowed similarities and differences to be highlighted in stages 3 and 4 of the process, where data were indexed, charted and summarised. The perspectives of patients, family members and HCPs are given individually below, before commonalities and divergences between the three groups are discussed.

#### Patient perspectives

In addition to giving feedback about the Patient Dignity Question, patients made many comments about their care, which fell into eight subthemes. These eight subthemes were grouped into three main themes: Attributes and attitudes, Know me as a person, and Time and place. Details are given in Table 9 below.

**Table 9:** **Themes and subthemes: patient perspective**

**Theme**	**Subthemes**	**Details**	**Verbatim examples**
**Attributes and Attitudes**	● Appreciation	Appreciation of staff attributes;	***“I appreciate the whole team… people try… they can’t do enough for you” P.23***
	● Staff attitudes	attitudes of staff towards patient	***“It was just the way they went about the whole thing, telling me ‘sorry, you’re dying’***
			***…and ‘we’ll refer you to palliative care….***
			***you’re not priority, we will never operate on you’…***
			***I felt like a second class citizen” P. 11***
**Know me as a person**	● Knowledge of person	Knowledge of the patient as an individual person,	***“I was’nae treated as a person, I was treated as somebody that was in a bed” P.11***
	● Emotional awareness	including their life achievements; knowledge of	***“*** **(Staff need to know)** ***that***
	● Personal achievements	people who can act in an advocacy role on patient’s	***I am terrified of dying. I have a fear of death being painful, a bad experience” P. 29***
	● Advocacy	behalf, or those people patient wants to protect. Use of the PDQ in achieving this	***“*** **(I was)** ***a chartered mechanical engineer, with a first class honours degree” P. 2***
			***“I must get*** **(family member)** ***sorted out. They can throw me in the back green, but I must get*** **(family member)** ***sorted out” P. 15***
**Time and place**	● Staff time	Staff time and organisational structure: micro and macro structures. Adherence to structural regimes	***“..Lack of communication is a big thing for me…you know they, they wander about there and nobody comes across and speaks to you” P. 18***
	● Adherence		***“Staff speak about ward being short staffed, how busy they are etc.…***
			***they don’t really speak to me about anything else” P. 14***
			***“I struggle with the time and speed things happen in hospital, like the routine in the morning” P.16***

Comments and feedback made by healthcare professions fell into two broad categories: Care and communication and enlightenment and emotions (see Table 10 for details).

**Table 10:** **Themes and subthemes: HCP perspective**

**Theme**	**Subtheme**	**Details**	**Verbatim examples**
**Care & communication**	Care from the HCP perspective	Knowledge about impact on/of care; communication as a connection tool	***“Formal written wishes are a good idea” HCP P. 14***
			***“I’ve tried to keep the*** **(PDQ)** ***summary in mind when speaking to the patient” HCP P. 16***
**Enlightenment and emotions**	New knowledge and emotional response	Knowledge about patient as a person, and associated emotions	***“*** **(The PDQ)** ***allows staff to get a bit of insight into the patient that is not always obvious when first meeting the patient” HCP P. 5***
			***“I didn’t know she felt so strongly about not being thought of as a ‘cancer patient’” HCP P. 16***
			***“I feel I understand this better, as I know more of his situation and how it makes him feel” HCP P. 26***

#### Family member perspectives

There were fewer responses from family member participants, partly due the short time frame available, between asking the PDQ question, displaying the PDQ response and getting feedback. The short time frame was also due to patients getting sicker, being discharged to home, hospice or care home and being transferred to other wards. As well as, the practicalities of family members being available during that time. This is, however, understandable given that this was a pragmatic pilot study. Nevertheless, it is useful to indicate the responses of family members who did take part, which fell into the two broad themes of Individualised care and Taking the time. See Table 11 for details.

**Table 11:** **Themes and subthemes: family member perspective**

**Theme**	**Subtheme**	**Details**	**Verbatim examples**
**Individualised care**	Using the PDQ to improve care	Family members felt the PDQ improved care, and helped the patient be treated as an individual	***“I looked at this*** **(PDQ response)** ***and thought ‘this is you’, so knowing this would help staff to have an idea*** **…(I’m)** ***happy for anything that improves care for people – I think this might” FM P. 20***
			***“*** **(The PDQ)** ***is feedback from patients who are in the worst possible circumstances really, and their view obviously is the one that matters” FM P. 30***
**Taking the time**	Staffing levels inhibit interaction	Staffing levels and other duties reduced the time available to talk to patients	***“*** **(The nurses)** ***are plagued with paperwork; it deters them from spending time with the patients” FM P.29***
			***“The staffing levels aren’t good; I know they do the best they can, and I know there’s considerable restriction on finances” FM P. 30***

### Common themes across perspectives

Once indexing and charting of individual perspectives had taken place, reduction of material enabled brief but understandable summaries of what was said by all participants possible, at the same time as continuing to link summaries to transcripts. Mapping and interpretation then allowed for merging of the individual perspectives into over-arching themes, and a common understanding that concurred across participants. See Table 12 for details.

**Table 12:** **Derivation of over-arching themes**

**Over-arching themes**	**Themes (and attribution)**	**Subthemes (and attribution)**
**Attitude and Approach**	Attributes and attitudes (Pts)	Appreciation (Pts)
	Care and communication (HCPs)	Staff attitudes (Pts)
		Care from the HCPs perspective (HCPs)
**Patient as Person**	Know me as a person (Pts)	Knowledge of person (Pts)
	Individualised care (FMs)	Emotional awareness (Pts)
	Enlightenment and emotions (HCPs)	Personal achievements (Pts)
		Using the PDQ to improve care (FMs)
		New knowledge and emotional response (HCPs)
**Taking time**	Time and place (Pts)	Staff time (Pts)
	Taking the time (FMs)	Adherence (Pts)
		Staffing levels inhibit interaction (FMs)

Pts = patients; FMs = family members; HCPs = Healthcare professionals.

The shared perspectives related to: individualised care, and knowing the patient as a person (*patient as person*); adopting the right attitude and approach to care (*attitude and approach*), and having the time to talk and implement care (*taking time*). Although restrictions on staff time were highlighted by patients and family members, this aspect of care was rarely referred to by HCPs.

#### The patient dignity question

Comments regarding the use of the PDQ were also pooled across all participants.

It was generally accepted by the three groups of participants that the PDQ was a useful tool in helping to facilitate knowledge of the patient as an individual; such knowledge assisted understanding and empathy. There were many positive responses from patients with regard to the use of the PDQ in promoting person-centred care, for example:

***“It empowers you to feel like you’re putting something in as well as somebody else” P.11***

Not everyone felt the PDQ would improve the care they received, because it was already considered good ***(“Just the tops” P. 21)***; one person commented on the benefit of having it completed on the first day of hospital admission.

From the results, the majority of HCPs felt the PDQ told them something new about the patient in their care; where HCPs did not feel they learnt something new about their patient (n = 5; 29.4%), the PDQ did increase feelings of connection or empathy:

***“I feel I understand this better, as I know more of his situation and how it makes him feel” HCP P. 26***

It also increased feelings of connectedness, and confirmed that ***“We are doing a good job” HCP P. 1.***

Only one fairly newly qualified HCP did not read the PDQ, and felt her ***“standard of professionalism would not be changed in anyway” (HCP P. 27)****.* However, without reading the PDQ and patient response, it might have been difficult for her to make this assessment with any degree of accuracy.

## Discussion

The findings of this pilot study provide understanding into the provision of care that treats people with dignity, and as individuals - core principles of the basic human rights of patients [17]. These will help in the development of a larger scale study which we anticipate will have important implications for clinical practice. These pilot study results suggest that the PDQ has the ability to make improvements to two elements of care as perceived by patients: the person-centred environment *and* empathetic care. This is supported by the strong positive correlation between higher PCQ-P and CARE scores post PDQ.

The dispersion of PCQ-P summated scores suggests that the PDQ intervention led to improved patient-centred care, as rated by patients. On the other hand, there do also appear to be higher frequencies of lower end scores post PDQ intervention. This suggests that the PDQ intervention may require improvement in order to capture higher ratings in terms of perceived patient-centred climate. However, overall, scores at the higher end have greater frequencies post-PDQ, suggesting a general increase in patient satisfaction with perceived care. This is promising when considering the PDQ as an intervention to benefit patient’s sense of dignity in palliative care settings.

Dispersion of CARE summated scores revealed similar trends. The greater frequency of higher CARE scores supports the PDQ as an intervention that benefits patients’ perception of empathy in palliative care settings. The lack of statistical significance from the Wilcoxon Signed Rank Test carried out on pre and post intervention summated PCQ-P and CARE scores could be accounted for by the small sample of the study [18]. Furthermore, the lack of normal distribution made non-parametric techniques more appropriate and these are recognised as being less sensitive and not as powerful as parametric tests [16]. Therefore, genuine statistical significances may go undetected. However, obtaining statistical significance between different scores from two time periods does not necessarily prove that the intervention was accountable for these differences; rather it gives a possible indication. With regards to the data in this study, although statistical significance was not obtained, the descriptive statistics do show that the median score was increased for PCQ-P post PDQ. The scores at the lower and upper quartiles of data for both PCQ-P and CARE increased post-PDQ. Therefore further exploration of the effectiveness of the PDQ intervention would be beneficial.

The effectiveness of the PDQ intervention is supported by the fact that the minimum CARE summated score increased by 5 post-PDQ. The maximum summated score could not increase post-PDQ, since the highest score available was 50. The fact that summated scores were high pre-PDQ may suggest that the HCPs in this study were already giving a good level of empathetic, person-centred care. However the findings do suggest that the PDQ improved patient-care, since descriptive statistical analysis shows that the minimum CARE score had increased post-PDQ. For the PCQ-P patient outcome, both the minimum and maximum summated scores increased. Promisingly, the maximum summated PCQ-P score was 101 out of a maximum of 102. This demonstrates an extremely high level of patient-centred care according to the PCQ-P patient outcome measure after the PDQ intervention had been introduced.

Higher PCQ-P scores post-PDQ were associated with higher CARE scores post PDQ, as confirmed by the results from Spearman’s rho, suggesting that when the PDQ has positive effects as perceived for the patient, it resonates with two areas of patient care - the patient-centred climate and patient’s perception of empathy. Both these patient-centred outcomes are worth considering for future study of the PDQ intervention.

The results also show that the PDQ may support the disclosure of previously unknown information that may have a bearing on clinical decision making, thereby increasing the chance of individual wishes being incorporated into care that is more person-centred as a result [19]. This was considered to be important affirmation of the use and relevance of the PDQ to clinical practice.

All patients completed the PDQ themselves, and the information divulged was re-inforced by relatives, where they took part. This was deemed notable, because relatives often act as proxies in cases where the patient has lost the ability to convey wishes as disease processes progress. From the PPI and PPS scores, few patients were expected to die within 6 weeks of the research taking place, so they were able to voice their opinions themselves at this stage.

As displayed in Table 7, within our limited sample, all patients agreed that the information was accurate (100%), important for health providers to know (83.3%), and something they would recommend to others (93.3%). This was considered a valuable endorsement of acceptability. The fact that all participants were agreeable to the information being visible and shared with healthcare staff validates the fact that they felt it was important and relevant to their care, and provided information they felt appropriate to disseminate.

For many of the items on the PCQ-P and CARE questionnaires, more patients showed an increase in scores, rather than a decrease. However, it should also be noted that many of the patients reported no change post-PDQ regarding PCQ-P and CARE outcomes, which might indicate that some improvements could be made to the use of the PDQ. Data from a larger sample will be useful, particularly with regards to carrying out more inferential statistical tests. For example, cross-tabulations showed general improvement in patients’ scores for both PCQ-P and CARE items and the HCPs’ data revealed that, generally, respondents agreed that the PDQ had produced an effect on the eight items measured. However, when inferential statistics were applied using summated scores for both patients and HCPs, no significant differences were derived. As mentioned earlier, this is probably a consequence of the small sample of participants, which is a well-recognised limitation in failing to achieve statistical significance [16], particularly in pilot studies.

Despite the absence of statistical significance, health care professionals nevertheless reported improvements in their attitude, empathy and connectedness with the patients as a result of reading their brief PDQ response. Table 8 shows that for all items, the majority of HCPs agreed or strongly agreed that the PDQ had enhanced some aspect of their care in terms of influence on attitude, empathy, connectedness and care satisfaction. Fifty percent of HCPs strongly agreed or agreed that the PDQ had influenced their care of the patient. Only a very small minority selected “strongly disagree” or “disagree” for items on this questionnaire. This concurs with the data indicating that over half of patients believed that the PDQ would affect the way that the HCP would give care (63.3%).

Patient and family responses during interviews indicated that staff attitudes and approaches were a very important part of feeling respected as an individual. Increasing workload demands, patients with more complex health problems, changing population demographics, and the need to adapt to new technology, can all contribute to staff stress and difficulties, particularly for those working in the acute in-patient sector [20]. The PDQ may provide an effective means of positively influencing staff attitudes by acting to reinforce personhood.

Caring for people who are at end of life also requires a particular skill set that may prove challenging for staff, particularly those with limited experience [21]. Of the HCPs included in this study, 62% had no prior palliative care experience. Support for professional development, and understanding of the emotional impact of caring on staff, do need to be explicitly built into healthcare systems, if poor care or attitudes are to be avoided [22].

Both patients and relatives highlighted the importance of staff having sufficient time to talk. Recent initiatives [23] encourage the facilitation of procedural and structural changes within the immediate care environment that promote more effective and efficient working environments. However, fewer than 50% of acute care teams in this research study area had managed to access this training at the time of data gathering [23], which may have explained some of the observations made by patients and family members with regard to staff being too busy to talk. New initiatives may take time to filter down and become embedded in practice at local level; however, this is necessary if hospital wards are to be prevented from being ‘toxic’ rather than healing environments [24].

If patients’ needs for support are ignored or not acknowledged, as noted in some of the comments from our participants above, this may act as a threat to dignity. Acknowledgement of patients’ support needs is highlighted by the MacMillan Values Based Standard™ [25]. Additionally, recognition that undignified care can result from inadequate staffing levels [26], has resulted in measures to ensure that appropriate care is more easily provided when adequate staff numbers are maintained.

A knowledgeable proxy can prove invaluable to ensuring the wishes of the patient are still paramount and can be communicated, even when they are unable to adequately express these themselves [19]. The PDQ can provide guidance regarding patient views on how they wish to be treated in the future, noted at a time when they are still able to communicate these wishes themselves.

Our results show that communication between staff and patients would benefit from improvement. Lack of, or poor, communication is not confined to acute hospital areas [27]; however, sensitive communication, given in a way that respects privacy, may be more of a challenge in the acute sector due to the very environment in which it takes place [25]. Overcoming these challenges can be enabled by taking an approach that seeks to understand the patient as a person; within this pilot study the PDQ has been shown to facilitate this.

### Strengths and limitations

A strength of this study is the fact that we were able to include the views of patients, professionals, and family members. Participants were recruited within a relatively short time span, and at a vulnerable time in their lives, which may have impacted on their responses. Due to time restraints and availability, fewer family members than anticipated were able to participate, which may have limited the findings from their perspective. The overall small sample of respondents across all groups (patients, HCPs and family members) limited some of the quantitative exploration. Nevertheless, quantitative analysis has suggested that that PDQ provides improvements as perceived by patients for key elements of care in terms of patient-centred aspects and empathy. Moreover, for HCPs, results show clearly that the majority agreed that the PDQ had enhanced their attitude, care, respect, empathy, connectedness and satisfaction in caring for the patient. This provides an incentive to develop the study further with a larger sample of respondents. The overall results of this mixed methods pilot study have provided understanding into patient, family and HCP perspective of the PDQ and will help develop the study into a larger multi-site trial.

## Conclusions

In relation to the primary aim and hypothesis regarding the effectiveness of the PDQ, the results have shown that patient feedback from the PCQ-P and CARE measure indicated small overall improvements in the majority of fields. The summated median score for PCQ-P increased post-PDQ. Although an increase in the median CARE score post PDQ did not increase, the fact that it did not decrease is positive. Furthermore for both patient outcomes, the minimum summated score increased post PDQ and increases are also evident for the lower and upper quartiles of the data. This provides initial evidence to support further study to measure the effects of PDQ on a larger sample of patients. This is further encouraged by the fact that over half of patients believed that the PDQ would change the way HCPs cared for them and that 93.3% would recommend the intervention to others. HCP data showed positive enhancement to several aspects of caregiving, with many items receiving a “strongly agree” or “agree” rating.

With regard to the secondary hypothesis about the acceptability of the PDQ in clinical use for patients with palliative care needs in the acute sector, results have shown it was well received by patients, family members and staff.

This pilot study has therefore shown that the PDQ has the potential to be a valuable and acceptable tool in the provision of person-centred care. It can provide information that may not be available through routine processes and procedures. We conclude that the PDQ could be incorporated into clinical practice for the care of palliative care patients in the acute setting to the benefit of personalized and dignified care.

Further research using the PDQ across a wider geographical area, and in a more diverse number of settings, will be beneficial.

## Additional file

Additional file 1:
**Abbreviations.**
