# Supplementary material for: The dignified approach to care: a pilot study using the patient dignity question as an intervention to enhance dignity and person-centred care for people with palliative care needs in the acute hospital setting
Source: BMC Palliat Care. 2015 Apr 9;14:9. doi: 10.1186/s12904-015-0013-3 (PMC4399754; doi:10.1186/s12904-015-0013-3)
Supplement: Additional file 1: — Abbreviations. [file 12904_2015_13_MOESM1_ESM.docx]

**Additional file 1**

**Abbreviations**

| CARE | Consultation and Relational Empathy measure |
| --- | --- |
| HCP | Health Care Professional |
| PCQ-P | Person-centred climate questionnaire- Patient version |
| PDQ | Patient Dignity Question |
| PPI | Palliative Prognostic Index |
| PPS | Palliative Performance Scale |
| PRS | PDQ Responsiveness Score |
| R&D | Research and Development |
| SPSS | Statistical Package for the Social Sciences |
| UK | United Kingdom |
